# Effect of dietary supplementation of a phytogenic blend containing *Schisandra chinensis*, *Pinus densiflora*, and *Allium tuberosum* on productivity, egg quality, and health parameters in laying hens

**DOI:** 10.5713/ajas.20.0552

**Published:** 2020-11-09

**Authors:** Seung-Gyu Moon, Sung-Kwang Lee, Woo-Do Lee, Kai-Min Niu, Won-Uk Hwang, Jong-Seok Oh, Damini Kothari, Soo-Ki Kim

**Affiliations:** 1 Department of Animal Science and Technology, Konkuk University, Seoul 05029, Korea; 2 Institute of Biological Resource, Jiangxi Academy of Sciences, Nanchang 330029, China

**Keywords:** Laying Hens, Fermented *Schisandra chinensis* Pomace, *Pinus densiflora* Needle Extract, *Allium tuberosum*, Feed Additive

## Abstract

**Objective:**

This study was conducted to investigate the supplementary effect of a phytogenic blend (SPA: a mixture containing fermented *Schisandra chinensis* pomace, fermented *Pinus densiflora* needle extract, and *Allium tuberosum* powder in the ratio of 2:2:1) on egg production, egg quality, blood constituents, and visceral organs in laying hens.

**Methods:**

A total of 135 Hy-line brown laying hens (48-wk-old) were randomly allocated to three dietary treatments with 5 replicates of 9 hens. The control group (CON) was fed a basal diet (no exogenous SPA addition) and the experimental groups were fed the basal diet containing SPA at the level of 0.1% and 0.3% for 6 weeks.

**Results:**

The feed intake was significantly improved in SPA supplemented groups as compared with the control (p<0.05). However, egg production, daily egg mass, and feed conversion ratio were not different among the dietary treatments (p>0.05). For egg quality traits, only Haugh unit (HU) was significantly improved in SPA (0.3%) (p<0.05) as compared with other groups. However, HU was not affected during 4-wk of storage at 18°C among the dietary treatments (p>0.05). Furthermore, SPA supplementation did not affect the blood biochemical constituents except for the phosphate content, which was significantly higher in SPA groups than the CON group (p<0.05). There were no significant differences in visceral organ characteristics and immune indicators (immunoglobulin A [IgA], IgG, and IgM) in SPA or CON groups.

**Conclusion:**

This study suggested that the supplementation of SPA may have beneficial effects on feed intake and egg quality in laying hens.

## INTRODUCTION

Antibiotics have been routinely used in farm animal production since mid-1950s to promote their well-being and growth. However, the prolonged and unregulated use of antibiotics have raised a concern of the emergence of antibiotic resistance in the animals’ bacteria and from there to bacteria infecting humans [1]. The poultry industry has grown substantially in the direction of improving the productivity with the use of alternative antibiotics to improve growth and feed conversion efficiency as well as to prevent infections [2]. In this context, phytogenic feed additives (PFA) comprising a wide variety of herbs, spices, and plant-derived extract have received considerable attention in poultry diets over other commonly used non-antibiotic growth promoters, such as organic acids and probiotics due to their safe and environmentally friendly nature [3–6]. PFA contain several bioactive compounds such as polyphenols, essential oils, saponins, and terpenoids [7], which are reported to have digestion stimulating effects [3], antimicrobial and antioxidant properties [5], and anti-inflammatory potential [8]. Several recent studies indicated that the dietary supplementation of PFA improves productive performance, egg quality, digestibility of nutrients, some blood biochemical constituents, and immune activity in laying hens [3–6,8]. However, the efficacy of these additives is influenced by several factors such as plant species, extract composition, application method and levels, poultry age and breed, and housing conditions [3,9].

*Schisandra chinensis* (*S. chinensis*) Baill. of Schisandraceae family is native to Far East of Russia, South of China, and Korea [10]. The fruit of *S. chinensis*, commonly called as a “five-flavored berry”, is rich in lignans, with dibenzocylooctadiene lignans (type A) being the most abundant (80%). In addition, the berries are rich in phytoestrogens, minerals, vitamins, and essential oils [11]. Research on *Schisandra* fruit revealed that its active lignan possesses antioxidant, antimicrobial, hepatoprotective, and anti-inflammatory functions [10,12]. The inclusion of *Schisandra* meal in broiler diets was shown to improve meat quality [13] and some blood antioxidant parameters [14].

Pine needles have been traditionally used as supplements in various foods and folk medicine in Asia, specifically in Korea and China [15]. Pine needles exhibit several biological activities, including antimicrobial, antioxidant, antimutagenic, antitumor, and anticholesterol [15–17]. These beneficial properties may be related to their bioactive compounds such as phenolics, flavonoids, and tannins [17]. Recently, several studies indicated the potential of pine needles as natural feed additives in poultry production via their stimulatory effects on the antioxidant status of birds [16,18–21]. However, it was also indicated that the high content of condensed tannins in pine needles might affect the nutrient absorption and protein digestibility in animals [22].

Chinese chive (*Allium tuberosum*) is widely used for culinary purposes and its application in folk medicine has also increased its popularity in Asia [23]. Chinese chive has been reported to demonstrate antioxidant [23], antimicrobial [24], hepatoprotective [25], and anti-inflammatory [23] activities. The beneficial properties of Chinese chive are attributed mainly to organosulfur compounds, polyphenols, and saponins [26].

To our knowledge, little research reported on the effect of these additives in laying hens. Therefore, the current study was designed to evaluate the effect of *Schisandra* fruit pomace, pine needles, and Chinese chive supplementation to layer diets on laying performance, egg quality, egg storage, blood constituents, and visceral organs.

## MATERIALS AND METHODS

### Preparation of feed additive

A phytogenic blend was prepared by mixing fermented *S. chinensis* pomace, fermented pine needle extract, and Chinese chive powder in the ratio of 2:2:1 (SPA) based on the preliminary experiments. The *S. chinensis* fruit pomace was procured from a juice-making plant (Mungyeong-si, Gyeongsangbuk-do, Korea), sun-dried for 24 to 48 h and stored at 4°C until use. The fruit pomace was then fermented using an indigenous isolate, *Wickerhamomyces* sp. SK1819 (1%, v/v) in yeast malt broth (Difco Laboratories, Detroit, MI, USA) at 1:1 ratio at 30°C and 100 rpm for 16 h. The fresh pine needles (*Pinus densiflora*) were collected from the region of Bonghwa-gun, Gyeongsangbuk-do, Korea. The needles were then washed, air-dried, and spontaneously fermented in a medium containing equal amounts of water and sugar for a period of 1 year. The supernatant of this spontaneously fermented pine needles was used as a fermented pine needle extract. The Chinese chive was purchased from a local market (Seoul, Korea) and juice was squeezed using a juicer (Angel-juicer, Busan, Korea). Chinese chive powder was prepared by mixing its juice with soybean meal (used as excipient) in the ratio of 7:3 and dried at 40°C for 36 h in a dry oven (WFO-600S, Eyela Singapore Pte. Ltd., Singapore). Table 1 presents the proximate chemical composition of the tested SPA, including moisture, crude protein, crude fat, crude fiber, ash, calcium, and available phosphorus [27].

### Experimental animal and design

A total of 135 Hy-line brown laying hens was randomly assigned to 3 treatment groups with 5 replicates of 9 birds each. The experimental house was a 2-tier battery-cage facility and hens were housed 3 per cage of the dimension of 43 cm length, 45 cm deep, and 42 cm height. A corn-soybean meal basal diet was formulated to meet or exceed the 1994 National Research Council recommendations [28] (Table 2). The levels of SPA supplied to the basal diet was as follows: 0%, 0.1%, and 0.3%. The appropriate amount of SPA was added to the basal diet and mixed for 5 min using a feed mixer (DKM-350SU, Daekwang Co., Ltd., Hwaseong, Gyeonggi-do, Korea) to obtain a homogeneous mixture. The feeding experiment was performed for 6 wk after 2-wk of adaptation period. During the entire experimental period, *ad libitum* feed in mash form and water were provided with 16 h light and 8 h of a dark period.

This experiment was performed in accordance with the Animal Care and Use Committee (KU18129) of Konkuk University (Seoul, Korea) in November 2014.

### Egg productivity

The number of eggs produced was recorded daily at 13:00 h including those were broken. Egg production rate (%) was calculated from the total number of eggs laid in 1 wk divided by the total number of hen days in that week on a replicate basis. Average egg weight was obtained by dividing the total weight of collected eggs by the number of normal eggs. The daily egg mass was calculated by multiplying the egg production rate by the average egg weight. We recorded feed intake weekly for each replicate. The feed conversion ratio (FCR) was calculated as grams of feed intake per gram of daily egg mass produced.

### Egg quality

On a weekly basis, fifteen eggs per treatment (3 eggs per replicate) were randomly collected for the egg quality measurements including egg weight, egg breaking strength, Haugh unit (HU), eggshell color, yolk color, and eggshell thickness. The egg breaking strength was measured using egg breaking strength tester (FHK, Fujihira Co. Ltd., Tokyo, Japan). HU, a measure of the height of the albumen of the eggs broken out on a flat surface, was calculated using the formula 100×log (H+7.57–1.7W0.37), where H is the height of the egg white (mm) and W is the weight of the egg (g). Egg shell color was measured using an eggshell color fan (Samyang Co., Ltd., Seoul, Korea). Egg yolk color was measured using an egg yolk color fan of Roche. Egg shell thickness was measured at central part of the eggshell fragments without eggshell membrane using a Digimatic micrometer (Series 293-330-30, Mitutoyo Corporation, Kawasaki, Japan).

### Egg storage

Four days before the end of the experiment, all the normal eggs were collected to measure the HU according to the storage period of the eggs. Collected eggs were stored at 18°C for 4 wk, and a total of 45 eggs, 3 eggs with similar egg weight per replicate, were selected at 1, 2, and 4 wk to measure the HU as described above.

### Blood parameters

After 6 wk of feeding, 8 hens were selected from each treatment with a standard weight of 2.1±0.1 kg to measure the blood parameters. About 10 mL of blood was collected from the carotid artery and serum was separated by centrifugation at 1,500 rpm for 20 min. The separated serum was sent to Seoul Clinical Laboratories (Giheung-gu, Yongin-si, Gyeonggi-do, Korea) to analyze the levels of aspartate aminotransferase (AST), alanine aminotransferase (ALT), triglyceride (TG), total cholesterol (TC), albumin, globulin, creatinine, calcium, phosphate, amylase. The analysis was performed with an automated analyzer (Hitachi 7600, Tokyo, Japan). High-density lipoprotein (HDL) cholesterol was measured using an HDL diagnostic kit (HDL-cholesterol kit, Youngdong Medical Corporation, Seoul, Korea). Afterthat, low-density lipoprotein (LDL) and very-low-density lipoprotein (VLDL) cholesterol were precipitated by adding sedimentation reagent (dextran sulfate, phosphotungstic acid) to the serum and the remaining ester-type cholesterol was decomposed with esterase and released. Subsequently, an enzyme solution (cholesterol oxidase, peroxidase) was added to form a red quinoid pigment, and the absorbance was measured at 500 nm. HDL (%) represents the percentage of HDL in TC content, and LDL+VLDL value was calculated by subtracting HDL from TC.

### IgA, IgG, and IgM contents in the blood

Blood immunoglobin (Ig) including IgA, IgG, IgM content was measured using the Chicken IgA, IgG, and IgM ELISA Kit (Bethyl Laboratories, Inc., Montgomery, TX, USA). The prepared samples and standards were placed in a chicken IgA pre-coated 96-well plate 100 μL each and left at room temperature for 1 h. It was then washed four times with a wash solution (50 mM Tris, 0.14 M NaCl, and 0.05% (v/v) tween 20). Then, 100 μL of chicken IgA, IgG, and IgM detection antibodies were added to each well, and then reacted at room temperature for 1 h, and washed again four times using a washing solution. Subsequently, 100 μL of antibody Avidin-horseradish peroxidase conjugated solution was added to each well, followed by reaction for 30 min, and then washed four times. Then, 100 μL of tetramethylbenzidine substrate solution was added to each well, and color change was observed for 30 min in the dark. Finally, the reaction was stopped by adding 100 μL of stop solution (2M, H_2_SO_4_) to each well, then absorbance was measured at 450 nm using a microplate reader (Benchma plus, Bio-Rad Laboratories, Hercules, CA, USA). Standard curves were used to calculate the content of IgA, IgG, and IgM in blood.

### Visceral organ properties

After collecting blood, liver, spleen, gizzard, heart, abdominal fat, jejunum, ileum, cecum and ovary were collected and expressed in terms of relative weight per 100 g of body weight. The length of jejunum and ileum were measured after removing the mesentery and expressed as relative length per 100 g of body weight. After that, the contents of jejunum and ileum were collected, and the pH of the contents was measured using a digital pH meter (735P, iStek Inc., Seoul, Korea).

### Statistical analysis

Data are analyzed in a completely randomized design using the PROC mixed procedure of SAS version 9.4 (SAS Institute, Cary, NC, USA). The data of productivity performance and egg quality were analyzed by considering replicate as the experimental unit. For the measurements of blood and organ properties, individual bird was considered as the experimental unit. The significance of difference among the treatments was assessed using the Tukey’s test. Means were considered statistically different at p-value < 0.05. Results were presented as least squares means±standard error.

## RESULTS

### Effect on laying performance

The effects of dietary supplementation of SPA on laying performance in hens from 50 to 56 wk of age are shown in Table 3. During this period, the SPA supplementation into layers’ diet had no effects on egg production, egg weight, feed conversion, and egg mass when compared to the control group (p>0.05). However, a linear significant increase in the feed intake was observed with the increase in SPA levels (p = 0.03).

### Effect on egg quality

The effects of dietary supplementation of SPA in laying hens on HU, eggshell color, egg yolk color, eggshell breaking strength and eggshell thickness are shown in Table 4. Dietary treatments had a significant effect (p<0.05) on HU. The SPA (0.3%) group had higher HU as compared with control and SPA (0.1%) groups (p = 0.001). However, dietary supplementation of SPA had no effects on eggshell color, egg yolk color, eggshell breaking strength, and eggshell thickness (p>0.05).

### Haugh unit change according to egg storage period

**Figure 1:**
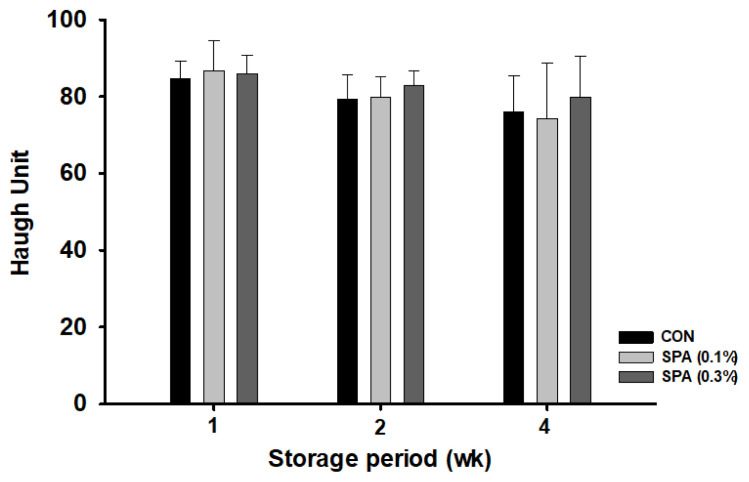
Effect of supplementary SPA on change in Haugh unit during egg storage of 28 d at 18°C. Values are presented as means±standard error. SPA, fermented *S. chinensis* fruit pomace, fermented pine needle extract, and Chinese chive powder in the ratio of 2:2:1. CON, control; basal diet; SPA (0.1%), basal diet+0.1% SPA; SPA (0.3%), basal diet+0.3% SPA.

The effect of dietary supplementation of SPA in laying hens on HU according to egg storage period is shown in Figure 1. During storage, the HU decreased in the case of all the dietary treatments. However, the supplementation of SPA had no effects on HU of eggs stored for 4 wk at 18°C (p>0.05).

### Impact on blood parameters

The effects of SPA inclusion in diets on blood biochemical parameters of layers at the end of the experiment (56 wk of age) are presented on Table 5. Serum levels of AST, ALT, TG, TC, HDL, HDL (%) LDL+VLDL, albumin, globulin, creatinine, calcium, and amylase did not differ among the dietary treatments (p>0.05). However, blood phosphate content was significantly higher in SPA supplemented groups than the control group (p = 0.02).

### Effect on immune markers in the blood

Table 6 shows the effects of dietary supplementation of SPA on laying hens on the Ig content in the blood. Ig including IgA, IgG, and IgM did not differ significantly in all the treatments (p>0.05).

### Impact on visceral organs properties

A summary of visceral organ weights of the experimental birds is shown in Table 7. After 6 wk of dietary SPA supplementation, there were no statistically significant changes in the relative weights of liver, spleen, heart, gastrointestinal tracts (gizzard, jejunum, ileum, and cecum), and abdominal fat in comparison to the control birds (p>0.05). The relative weight of the liver, however, was reduced (p = 0.09) in the supplemented treatment groups as compared with that of the control group. The pH and length of the jejunum and cecum remained unaffected among the dietary treatments (p>0.05) (Table 8).

## DISCUSSION

Several studies have supported the nutritional value of plant extracts as natural feed additives in layer diets [3–5,8]. In this study, egg production, egg weight, egg mass, and FCR were not affected with or without dietary supplementation of SPA, suggesting that there was no toxicity of the phytogenic used up to the level of 0.3 g/kg of diet. The absence of adverse effects on performance traits has also been reported in broiler breeds when fed with pine (*Pinus brutia*) needle powder (10 g/kg of diet) [18] and *S. chinensis* powder (5, 10, or 20 g/kg of diet) [13]. Alternatively, Kim et al [29] observed that the egg production rate was significantly improved when the mixtures of *Artemisia campestris* (*A. campestris*), *Camellia sinensis* (*C. sinensis*), *S. chinensis*, and *Viscum album var. coloratum* were fed to laying hens. Similarly, Ma et al [30] observed the beneficial effects of the *S. chinensis* on the egg production traits of laying hens during heat stress. The inconsistencies in these results could be related to the variation in animal breeds and their health status, dose of herbs, and bioactive constituents in the used plant extracts as well as the experimental conditions. In a concentration-dependent manner, the SPA used in this study improved the feed intake. It is assumed that SPA might have improved the palatability of feed due to their aromatic characteristics and thereby could promote feed consumption when added to layers’ diets. This result was consistent with previous reports those indicated increased feed intake in broilers fed pine (*Pinus yunnanensis*) needle powder at 50 g/kg diet [19] and onion (*Allium cepa*) powder at 30 g/kg diet [31]. Due to the non-availability of the information related to layers, the productive performance results of this study were also compared with the research that examined the supplemental effects of some PFA on the growth performance of broilers.

This study showed no significant difference in yolk color and eggshell properties (color, breaking strength, and thickness) among the dietary treatments. This is consistent with the previous reports that indicated no alteration in most of the egg quality traits in laying hens fed *S. chinensis* [32] and pine needles [33]. Li et al [32] reported that egg weight, eggshell thickness, egg white height, HU, and eggshell breaking strength had not changed in a study that fed 24-wk-old Lohmann Brown laying hens with 0.5% to 1% Chinese herbal mixture (containing 70% pine needles and 30% *Artemisia annua*) in the diet. Kim and Paik [33] also indicated no changes in eggshell strength, eggshell thickness, eggshell color, egg yolk, and HU when 66-wk-old Hy-line Brown hens were fed 0.2% plant extracts, including turmeric, *Angelica gigas*, *S. chinensis*, *Glycyrrhiza glabra*, and *Levisticum officinale*. On the other hand, Kim et al [29] demonstrated that HU and egg yolk color were significantly increased compared to control, but eggshell thickness, and eggshell breaking strength were not changed when a mixture of *A. campestris*, *C. sinensis*, *S. chinensis*, and *Viscum album var. coloratum* was fed to laying hens. The discrepancies in the results of egg quality may be ascribed to the variability in animal breed, age, dosage, and environmental conditions. The literature relating Chinese chive supplementation to layers is scarce. HU is the “gold standard” of internal egg quality determination. Eggs are graded based on their HU values: AA, 72 or more; A, 71 to 60; and B, <60 [34]. Herein, HU in all the dietary treatments were above 72, suggesting that the eggs produced in this study are of good quality. Moreover, the inclusion of SPA at 3 g/kg of diet in laying hen diets significantly increased HU. These improvements in HU may be attributed to the antioxidant property of bioactive constituents in *S. chinensis*, pine needles and Chinese chive. In particular, the antioxidant properties of these plant extracts have been reported in previous studies [14,18,23], which might have reduced the lipid and protein oxidation in eggs. In addition, the bio-active constituents of plants were shown to protect magnum and uterus, as well as enhance the albumen secretion in laying birds [35], however, this needs to be specifically studied. HU also acts an important indicator of egg freshness and it is related to shelf life [36]. In the current study, HU decreased with the storage time as expected, but the effect of dietary SPA inclusion was not observed in the HU of eggs during storage.

Blood biochemical constituents can be indicative of the health status of birds. In the current study, except serum phosphate levels, no changes in blood biochemistry were observed. Phosphorus is an essential nutrient for laying hens, which plays an important role in bone formation and maintenance, energy storage, cytoskeletal maintenance, and egg production [37]. The use of exogeneous phytase to improve the phosphorus availability and blood phosphorus content of monogastric animals has been shown to be an ideal approach and is actively studied [37,38]. However, few studies have been conducted on the effects of dietary plant extracts supplementation on the blood phosphorus content in laying hens. The dietary SPA used in this study resulted in an increase in serum phosphate, which might be caused due to the increased digestibility of phosphorus or due to the presence of higher amount of available phosphorus in SPA supplemented diets. The previous reports of Amad et al [39] and Hafeez et al [40] observed the improvement in the ileal digestibility of nutrients including phosphorus in broilers by a dietary PFA and attributed it to the stimulation of endogenous digestive enzymes and to an increased absorption surface area in the intestine.

The changes in size and structure of internal organs are important for predicting the effect of diet and its components on the development and function of organs in laying hens. Generally, as the size of the organ increases, the energy required to maintain the organ increases, which in turn decreases the amount of energy input to productivity [4]. Our results revealed that dietary SPA supplementation did not affect relative weights of internal organs (liver, spleen, heart, gizzard, jejunum, and cecum), intestinal length, intestine pH, and abdominal fat, which indicated that dietary supplementation with SPA up to 3 g/kg of diet could have no observed adverse effects on organ development. Research on the effects of supplementing PFA in layer diets on internal organ characteristics are limited.

Ig content plays an essential role in immune regulation. Three main classes of Ig exist in poultry: IgA, IgM, and IgY. IgY is a counterpart of mammalian IgG [41]. It has been thought that the antioxidant constituents of *S. chinensis*, pine needles, and Chinese chive might have a role in the development of immune response in birds. In this study, dietary treatments did not affect serum IgA, IgM, and IgG contents, suggesting no effects of SPA on humoral immune status of laying hens. In contrast, Ma et al [30] indicated that supplementation with *S. chinensis* at 10 g/kg of diet significantly elevated antibody responses against Newcastle disease virus in laying hens during heat stress. These inconsistent results could be explained by the fact that healthy poultry reared under clean and ideal environmental conditions usually do not respond to the feed additives [42]. Another explanation could be the higher dosage of SPA may be needed to stimulate humoral immune responses. Further studies need to be conducted in laying hens under stress conditions to evaluate the effects of SPA on immune responses.

In conclusion, dietary supplementation of SPA containing a mixture of fermented *S. chinensis* pomace, fermented pine needle extract, and Chinese chive powder, positively influenced feed intake, HU, and blood phosphorus in laying hens. However, most of the productivity and egg quality traits remained unaffected by SPA inclusion in layers’ diets. Moreover, no adverse effects on blood and organ characteristics were observed. Further research is required to assess the SPA effects on immune response indices under stress or pathogenic challenge conditions.

## Figures and Tables

**Table 1:** Proximate chemical composition of the tested SPA

Item	Amount (g/100 g)
Moisture	52.49
Crude protein	10.15
Crude fat	7.88
Crude fiber	7.56
Ash	1.74
Calcium	0.08
Available phosphorus	0.16

SPA, fermented *S. chinensis* fruit pomace, fermented pine needle extract, and Chinese chive powder in the ratio of 2:2:1.

**Table 2:** Ingredients and chemical compositions of the basal diet

Items	Amount
Ingredients (%)	
Corn	50.00
Corn dried distillers’ grains with solubles (DDGS)	21.24
Limestone	10.20
Soybean meal	7.42
Rapeseed meal	5.00
Sesame seed oil meal	2.00
Feather meal	1.50
Tallow	0.90
Mono dicalcium phospate (MDCP)	0.59
Syn. Lys-sulfate	0.38
Syn. Met (liq.)	0.11
Syn. Thr	0.03
Salt	0.20
Mineral premix1)	0.18
Vitamin premix2)	0.11
Sodium bicarbonate	0.10
Choline-chloride (50%)	0.05
Calculated values	
TMEn (kcal/kg)	2,800
Crude protein (%)	17.00
Crude fat (%)	5.30
Crude fiber (%)	3.70
Ca (%)	4.00
Lys (%)	0.83
TSAA (%)	0.72
Available P (%)	0.27

TMEn, true metabolizable energy; TSAA, total sulphur amino acid.

1) Mineral premix: provide followings per kg of diet: Fe, 30 mg; Zn, 25 mg; Mn, 20 mg; Cu, 5 mg; Co, 0.15 mg; Se, 0.1 mg.

2) Vitamin premix: provide followings per kg of diet: vitamin A, 40,000 IU; vitamin D_3_, 8,000 IU; vitamin E, 10 IU; nicotinic, 60 mg; pantothenic acid, 20 mg; vitamin B_1_, 4 mg; vitamin B_2_, 12 mg; vitamin B_6_, 6 mg; vitamin K_3_, 4 mg; folic acid, 2 mg; vitamin B_12_, 0.02 mg.

**Table 3:** Effect of supplementary SPA on productivity in laying hens

Item	Treatments1)			p-value

	CON	SPA (0.1%)	SPA (0.3%)	
Egg production (%)	81.25±6.94	89.62±1.21	84.09±7.02	0.10
Egg weight (g)	64.37±1.54	64.12±0.45	64.34±0.94	0.92
Egg mass (g/hen/d)	52.31±5.10	57.46±0.89	54.11±4.41	0.15
Feed intake (g/hen/d)	121.86±1.64^b^	123.63±1.14^ab^	124.08±0.68^a^	0.03
FCR (g feed/g egg)	2.39±0.23	2.18±0.04	2.36±0.21	0.19

Values are means±standard error.

SPA, fermented *S. chinensis* fruit pomace, fermented pine needle extract, and Chinese chive powder in the ratio of 2:2:1; FCR, feed conversion ratio.

1) CON, control, basal diet; SPA (0.1%), basal diet+0.1% SPA; SPA (0.3%), basal diet+0.3% SPA.

a,b Means within a same row with different letters differ significantly at p<0.05.

**Table 4:** Effect of supplementary SPA on egg quality in laying hens

Item	Treatments1)			p-value

	CON	SPA (0.1%)	SPA (0.3%)	
Haugh units	89.65±8.94^ab^	87.12±10.58^b^	92.20±8.04^a^	0.001
Egg yolk color	7.93±0.56	8.07±0.60	7.96±0.60	0.26
Eggshell color	11.68±1.46	11.70±1.38	11.84±1.34	0.68
Eggshell breaking strength (kg/cm^2^)	2.91±0.81	3.18±0.78	2.98±0.85	0.07
Eggshell thickness (mm)	0.41±0.04	0.41±0.05	0.81±3.86	0.39

Values are means±standard error.

SPA, fermented *S. chinensis* fruit pomace, fermented pine needle extract, and Chinese chive powder in the ratio of 2:2:1.

1) CON, control, basal diet; SPA (0.1%), basal diet+0.1% SPA; SPA (0.3%), basal diet+0.3% SPA.

a,b Means within a same row with different letters differ significantly at p<0.05.

**Table 5:** Effect of supplementary SPA on blood characteristics in laying hens

Item	Treatments1)			p-value

	CON	SPA (0.1%)	SPA (0.3%)	
AST (U/L)	150.00±7.39	151.75±9.74	162.40±11.26	0.17
ALT (U/L)	4.38±1.06	3.75±1.04	4.00±0.93	0.47
TG (mg/dL)	1,159.67±166.16	892.00±53.74	1,026.40±78.72	0.08
TC (mg/dL)	116.67±20.65	107.29±19.35	116.00±6.96	0.56
HDL (mg/dL)	11.58±3.56	13.75±6.45	11.08±0.74	0.57
HDL (%)	9.15±1.59	12.04±4.70	9.56±0.53	0.36
LDL+VLDL (mg/dL)	105.09±17.14	98.26±13.36	104.92±6.54	0.58
Albumin (g/dL)	2.70±0.19	2.66±0.23	2.6±0.23	0.77
Globulin (g/dL)	7.54±10.11	2.72±0.28	2.84±0.49	0.36
Creatinine (mg/dL)	0.26±0.08	0.28±0.06	0.25±0.09	0.84
Calcium (mg/dL)	20.48±2.17	21.56±1.60	20.60±1.59	0.60
Phosphate (mg/dL)	5.24±0.81^b^	6.96±0.68^a^	6.26±0.94^ab^	0.02
Amylase (U/L)	287.20±61.37	291.6±9.89	270.92±51.92	0.77

Values are means±standard error.

SPA, fermented *S. chinensis* fruit pomace, fermented pine needle extract, and Chinese chive powder in the ratio of 2:2:1; AST, aspartate aminotransferase; ALT, alanine aminotransferase; TG, triglyceride; TC, total cholesterol; HDL, high-density lipoprotein; LDL, low-density lipoprotein; VLDL, very-low-density lipoprotein.

1) CON, control, basal diet; SPA (0.1%), basal diet+0.1% SPA; SPA (0.3%), basal diet+0.3% SPA.

a,b Means within a same row with different letters differ significantly at p<0.05.

**Table 6:** Effect of supplementary SPA on immunoglobulins in laying hens

Item	Treatments1)			p-value

	CON	SPA (0.1%)	SPA (0.3%)	
IgA	189.18±7.46	190.46±13.75	190.31±10.68	0.98
IgG	37.33±14.62	33.79±14.19	29.77±14.34	0.67
IgM	35.96±5.19	46.19±8.82	45.69±14.31	0.18

Values are means±standard error.

SPA, fermented *S. chinensis* fruit pomace, fermented pine needle extract, and Chinese chive powder in the ratio of 2:2:1; Ig, immunoglobulin.

1) CON, control, basal diet; SPA (0.1%), basal diet+0.1% SPA; SPA (0.3%), basal diet+0.3% SPA.

**Table 7:** Effect of supplementary SPA on relative weights of organs in laying hens

Item	Treatments1)			p-value

	CON	SPA (0.1%)	SPA (0.3%)	
Liver	1.68±0.28	1.55±0.17	1.42±0.23	0.09
Spleen	0.10±0.02	0.09±0.02	0.10±0.02	0.94
Heart	0.43±0.08	0.42±0.06	0.45±0.06	0.61
Gizzard	1.52±0.12	1.61±0.22	1.59±0.10	0.46
Jejunum	0.71±0.13	0.61±0.17	0.66±0.10	0.37
Ileum	0.58±0.13	0.56±0.11	0.51±0.09	0.46
Cecum	0.29±0.05	0.27±0.04	0.27±0.06	0.70
Abdominal fat	4.63±1.26	3.77±1.09	4.35±0.84	0.29

Values are means±standard error.

SPA, fermented *S. chinensis* fruit pomace, fermented pine needle extract, and Chinese chive powder in the ratio of 2:2:1.

1) CON, control, basal diet; SPA (0.1%), basal diet+0.1% SPA; SPA (0.3%), basal diet+0.3% SPA.

**Table 8:** Effect of supplementary SPA on the length and pH of ileum and jejunum in laying hens

Item	Treatments1)			p-value

	CON	SPA (0.1%)	SPA (0.3%)	
Jejunum pH	5.90±0.17	6.08±0.27	5.99±0.15	0.25
Ileum pH	6.61±0.39	6.56±0.50	6.24±0.28	0.16
Jejunum length (cm)	69.44±3.70	62.38±5.18	65.25±8.12	0.08
Jejunum length/100 g BW	3.34±0.23	3.04±0.30	3.23±0.46	0.23
Ileum length (cm)	62.44±5.90	61.88±5.08	60.63±5.76	0.80
Ileum length/100 g BW	3.00±0.30	3.02±0.30	3.00±0.30	0.99

Values are means±standard error.

SPA, fermented *S. chinensis* fruit pomace, fermented pine needle extract, and Chinese chive powder in the ratio of 2:2:1; BW, body weight.

1) CON, control, basal diet; SPA (0.1%), basal diet+0.1% SPA; SPA (0.3%), basal diet+0.3% SPA.
